# Draft Genome Sequence of *Branchiibius* sp. NY16-3462-2, Isolated from a Mixed Clinical Sample

**DOI:** 10.1128/genomeA.00368-16

**Published:** 2016-05-12

**Authors:** Pascal Lapierre, Tanya A. Halse, Joseph Shea, Vincent E. Escuyer, Kimberlee A. Musser

**Affiliations:** a Bioinformatics Core, Wadsworth Center, New York State Department of Health, Albany, New York, USA; b Bacteriology Laboratory, Wadsworth Center, New York State Department of Health, Albany, New York, USA

## Abstract

Here, we report the release of a draft genome assembly of a Gram-positive cocci *Branchiibius* sp. NY16-3462-2 with a high-GC content, sequenced from a mixed clinical sample containing *Mycobacterium tuberculosis*. This genome is the first publicly available sequence from a representative of the genus *Branchiibius*.

## GENOME ANNOUNCEMENT

*Branchiibius* spp. are GC-rich, Gram-positive coccoid actinobacteria and are members of the *Dermacoccaceae* family. Little is known about the genus *Branchiibius*, with only a handful of sequences and publications regarding these species available in public databases. Only two species have been characterized so far, *Branchiibius hedensis*, isolated from a Japanese codling, and *Branchiibius cervicis*, isolated from the skin of patients with dermatitis (1, 2). Here, we present the first publically released genome of a member of the *Branchiibius* genus sequenced directly from a mixed clinical sample.

This genome was sequenced from an induced sputum of a patient infected with *Mycobacterium tuberculosis* (MTB) and incubated in a mycobacteria growth indicator tube (Bactec MGIT 960) to enrich for MTB complex. DNA extraction was performed as follows: An aliquot of the specimen was heat-treated at 80°C for 1 h and then frozen at −20°C. The samples were later thawed at 37°C and 1-mL pipetted into a 2-mL screw-cap tube, and pelleted by centrifugation at 15,000 rpm for 15 min. The supernatant was discarded and 200 µL of well-mixed InstaGene matrix (Bio-Rad) was added to the pellet using a pipette with a large bore tip. The sample was heated at 56°C for 30 min, and three sterile 3-mm-diameter glass beads were added to the tube. After vortexing for 10 s, the sample was heat-treated in a heat block at 100°C for 20 min and processed using the FastPrep-24 5G tissue homogenizer for two cycles of 6.0 m/s, each for 45 s. The Extracted DNA was then separated from the beads/matrix by centrifugation for 5 min at 15,000 rpm and pipetted into a new tube. DNA sequencing was carried out using the Illumina MiSeq platform. The sample library was prepared using a modified Nextera XT protocol, incorporating 15 PCR cycles during the indexing step. Genome assembly was performed directly on the raw reads using Spades version 3.7.0. with default parameters and the “--careful” option (3).

Genome sequencing of MTB directly from mycobacteria growth indicator tubes yields pure sequencing results in the vast majority of the cases, with a few exceptions where reads belonging to human flora, such as *Staphylococcus* sp. or *Streptococcus* sp., are sometimes recovered along with *M. tuberculosis* reads. However, in this case, more than half of the reads in the sample belonged to an organism that could not be initially identified at first using Kraken, due to the lack of homologous genome sequences in public databases (4). The contaminating organism was ultimately identified as a member of the genus *Branchiibius* by 16S rRNA searches*.* Contigs belonging to *Branchiibius* sp. NY16-3462-2 were binned based on a combination of sequence alignments against other MTB genomes and BLAST searches. The final assembly of *Branchiibius* sp. NY16-3462-2 has a total of 50 contigs (≥500 bp), with an *N*_50_ of 250,112, 67% average GC content, and a total assembly length of 3.84 Mb. The draft genome was annotated using the NCBI Prokaryotic Genome Annotation Pipeline (PGAP).

### Nucleotide sequence accession number.

This whole-genome shotgun project has been deposited at DDBJ/EMBL/GenBank under the accession number LVCF00000000. The version described in this paper is the first version.
